# Trust in pathways? Professionals’ sensemaking of care pathways in the Norwegian mental health services system

**DOI:** 10.1186/s12913-021-07424-x

**Published:** 2022-01-05

**Authors:** Tine Nesbø Tørseth, Marian Ådnanes

**Affiliations:** 1 The Mohn Centre for Innovation and Regional Development, Western Norway University of Applied Sciences, a Research and Competence Centre within the Field of Responsible Innovation, Bergen, Norway; 2 The University of Bergen, Bergen, Norway; 3 Department of Health Research, SINTEF Digital, Trondheim, Norway

**Keywords:** Care pathway, Sensemaking, Trust, Mental health; implementation

## Abstract

**Background:**

In January 2019, care pathways within specialist mental health and substance abuse treatment services were officially launched in Norway. The care pathway introduced timeframes for assessment and treatment, allowing a maximum of 6 weeks to finish assessment and provide the patient with a diagnosis, in addition to allowing a maximum of 6 weeks from diagnosis to the first evaluation. The different action points required coding. The system was based on goals to improve services by focusing on user participation, coordinated patient flow, avoidance of unnecessary waiting time, improvement of equal access to services regardless of geographic location, and increased emphasis on physical health and lifestyle.

The purpose of our study was to examine how mental health professionals made sense of care pathways and furthermore, how issues of trust affected the process of implementation.

**Methods:**

Our multiple case study included four outpatient clinics for adults in four community mental health centres (CMHCs) in different parts of Norway. Qualitative data were collected through in-depth individual and focus group interviews and analysed using systematic text condensation. The informants were treatment personnel and leaders in four different outpatient clinics for adults.

**Results:**

The results indicated four distinct themes or reactions to the care pathway and its implementation: 1) lack of clarity regarding the overall goals and content of the care pathway; 2) the increased burden of coding, registration and administrative work, which professionals experienced as a stressor; 3) an IT and medical record system that did not correspond to the coding of the care pathway; and 4) an unrealistic distinction between assessment and treatment. These themes/reactions increased the health professionals’ distrust towards the care pathway, and a process of *sensemaking* encouraged them to reduce the importance of the care pathway system and its implementation.

**Conclusion:**

Theories of trust help in understanding how mental health professionals interpret care pathway implementation. Distrust and resistance towards the care pathways overshadow some of the overall quality goals of the care pathway, a view that was indeed shared by mental health professionals.

## Background

In recent years, care pathways in health care have been developed at an increasing rate, and managers are expected to promote these pathways and their implementation in clinical work. However, research on care pathway implementation has shown that clinicians often have mixed or negative attitudes regarding the standardization of health care utilizing pathways [1, 2].

Mental health care is seen as a difficult service to manage in terms of implementing change and new innovations because of the strong professional values and identities by professionals working in this field [3, 4]. Furthermore, new guidelines such as care pathways often require new IT systems within hospitals; this need is portrayed as notoriously problematic because these systems interfere with health professionals’ usual workflows and because the anticipated benefits take time to materialize [5, 6].

The process of implementing care pathways for mental health services in Norway started in autumn 2018 with national and regional conferences to introduce the new system. A national plan for its implementation from 2018 to 2020 [7] was sent out to the regional health authorities. Organization of health care services through standardized care pathways (CPs) has occurred in several areas of Norwegian health care, with the implementation of CPs for cancer treatment as the largest national introduction of standardized service production [8]. The European Pathway Association (EPA) defines the standardization of care processes into CPs as “a methodology for the mutual decision making and organization of care for a well-defined group of patients during a well-defined period” [9]. The method defines goals and decision making, as well as the measures to include in the treatment. The measures should reflect evidence, best practice solutions, and the involvement of the patient [10, 11]. Schrijvers et al. (2012) [12] show how several definitions of care pathways have been used (e.g., “patient pathway”, “integrated care pathway”, and “clinical pathway”). Furthermore, the authors [12] recommend using the name and concept of “care pathways” from the EPA, and this convention is applied throughout this article.

In January 2019, the new guidelines within specialist mental health and substance abuse treatment services were officially launched in Norway, with treatment organized according to structured care pathways. The care pathway introduced timeframes for assessment, treatment and evaluation that did not exist previously. A maximum of six weeks is now allowed for patient assessment and diagnosis. The first follow-up evaluation is then required to take place within a maximum of six weeks after a patient’s assessment/diagnosis. The care pathway is guided by five strategic goals. These were increased user participation, increased collaboration and coordination, avoidance of unnecessary waiting time, improvement of equal access to services regardless of geographic location, and increased emphasis on physical health and lifestyle. To determine whether the time frames are followed and the overall goals achieved, several new “pathway codes” were introduced and are registered by the therapist or administrative staff. This registration allows the Directorate of Health to monitor development within the services.

Despite having clear strategic goals, the Directorate of Health was reticent about the execution of care pathways, leaving the interpretation and accomplishment to each hospital or unit within specialist mental health care.^1^ Furthermore, there was already much resistance towards the new care pathway from health professionals working in mental health services [13].

The process through which organizational actors attempt to explain, interpret and relate to new innovations or implementations has become a critically important topic in the study of organizations and is often theorized as sensemaking [14]. The ability of organizational actors to make sense of events or issues has been linked to change and its outcomes [15–17]. Trust is seen as fundamental for good-quality health care, new implementations, and outcomes in many national and local health care contexts [18–20].

To understand how trust emerges and influences care pathway implementation, we suggest that combining sensemaking theory with trust theory is a fruitful approach when analysing the complex implementation process. Sandberg and Tsoukas [21], in their review of 147 articles using sensemaking theory, report that only one article applied sensemaking in combination with trust theory and that there was a need for further research combining the two theories. In our study, we studied how health professionals who work in outpatient clinics made sense of care pathway implementation and how issues of trust affected this sensemaking.

*How do mental health professionals make sense of care pathways, and how do issues of trust affect the process of implementation?*

### Theoretical outlook

#### Understanding change: making sense of implementations

Sensemaking theory has been utilized in several studies examining change [22, 23] and implementation processes within the health care system [24–26].

A central element in much sensemaking research is an overall focus on the individual and the need to understand complex and confusing circumstances and turn them into comprehensible situations that enable purposeful action [10, 21, 22, 27, 28]. Sensemaking directs both cognitive and social mechanisms for coping with new or unexpected events, and it explains actors’ behaviour in practice [10, 24]. The experience of equivocality leads individuals to extract and interpret environmental cues through three sets of interweaving processes: perceiving cues (noticing), making interpretations and engaging in action [22, 23].

Sensemaking helps to resolve incongruity in ways that enable activity [22]. Moreover, individuals utilize sensemaking as a strategy when interpreting new innovations or change projects [21–23, 29, 30]. In recent years, repeated calls have been made to include materiality and relational practice in theory [21, 28, 31, 32]. The critical sensemaking perspective explains how sensemaking already exists in subjects, objects, values and practices when individuals understand and interpret the world from a specific role or identity. This approach comprehends sensemaking as a holistic practice where the context and environment are integral [21, 22, 31, 32].

#### Trust within mental health care

Trust plays an important role in relationships among the state, health care practitioners, and patients [33–36], and the meaning and enactment of trust is influenced by top-down policy-makers [37]. Gulati and Nickerson [38] define trust as the expectation that another organization can be relied on to fulfil its obligations, to behave in a predictable manner and to act and negotiate fairly even when the possibility of opportunism is present [38–40].

In Szulanski’s [41, 42] model on knowledge transfer and implementations in health care, the motivation of the source and credibility are important factors determining success or failure [41]. Furthermore, the same trust needs to exist among governmental agencies realizing national guidelines, health care services and the professionals involved [41]. For consideration of how trust affects these relationships, an assessment of the interests of the source, or trustor, is important [43]. This is in line with Sandstrøm et al.’s [2] research on the implementation of guidelines within mental health care, in which the authors conclude that *regardless of from whom guidelines are released, they are unlikely to be utilized or implemented in the care of patients if those further down in the hierarchy do not trust the source* [1].

When elaborating the role of trust within health care systems, one must be observant of institutions, the number of relationships that must be managed to deliver outcomes, and the importance of developing shared meanings to sustain delivery [43, 44].

#### Sensemaking and trust in mental health care: bridging the gap

Fuglsang and Jagd [45] examine how sensemaking may serve as a bridge between institutional contexts and interpersonal trust processes. The critical sensemaking perspective, introduced by Mills et al. [46] and elaborated further by Aaroma et al. [47], provides a framework for understanding how individuals make sense of their environments at a local level while acknowledging the societal context. By examining contexts, the critical sensemaking framework creates space for a discussion of how different policy implementations, such as care pathways, in which individuals operate affect the cues they extract and how they make sense of different events. Critical sensemaking positions the context as a link between dominant social values and individual action [46, 47].

Conceptualizing how trust influences sensemaking may be a useful way forward. Möllering [48] mentions three elements when explaining why trust depends less on the individual trustee and more on the social norms and values in which actions are embedded. The elements of familiarity, calculated interest, and compatible norms and values render trust [48]. Thus, enabling an understanding of trust means becoming familiar with these structures. One approach is to look towards these structures within the field of mental health care that exist during the time of care pathway implementation. The field of mental health care in Norway is heterogeneous, and different actors bring their own logic on how treatment should be organized and how the care pathway seeks to address these issues [13]. Scientific–bureaucratic medicine is a term from Harrison and Ahmad’s [49] research on care pathways and their guidelines and shows how doctors and psychiatrists relying on medication and evidence-based medicine could have a more positive outlook regarding care pathways than, for example, psychologists firmly believing in a trusted alliance between patients and professionals that is hard to standardize [13]. Despite these differences, a trend that has developed over the last decades is viewing different governance and policy arrangements, such as new public management and other standardized tools aimed at developing structures, policies and processes [50, 51], as mistrust of health professionals and a threat to professional value discretion and autonomy [9, 52, 53].

New policies therefore affect organization as much as they influence trust by impacting the identities, skills, and prioritizations performed by the professionals and managers [16, 54, 55].

In sensemaking, “individuals, drawing on identity resources, act on cues, influenced by trust, and enact new, sensible environments as they do so” [22]. This enables a context that affects which cues are extracted as well as the interpretation of the extracted cues [56].

**Fig. 1:**
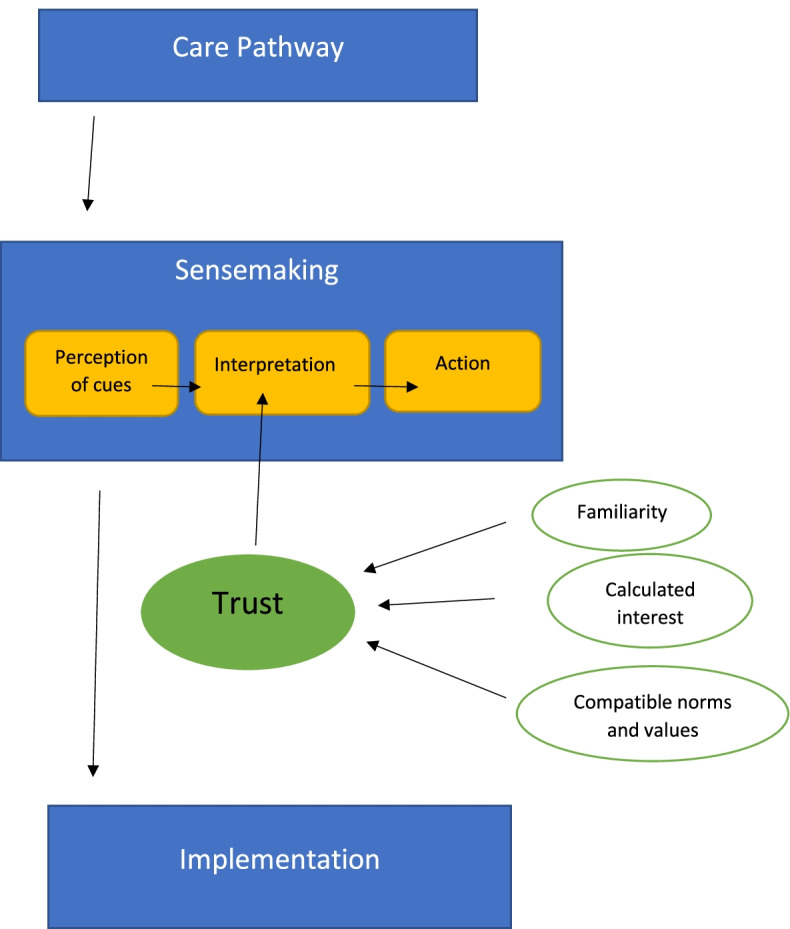
Trust and sensemaking

Analysing the outcome of care pathway implementation therefore means conceptualizing the theories presented in a more comprehensible framework. Such a framework is shown in Fig. 1, sensemaking and trust.

### Methodology

We used a multicase study design and qualitative techniques to understand the response of health professionals to care pathway implementation [57]. In this study, in-depth individual and focus group interviews with health professionals working in four different outpatient clinics for adults were performed. Qualitative interviews are a well-established and effective method of data collection and are particularly suitable for obtaining information on informants’ experiences and perceptions [49]. Focus groups provide a wide variety of data regarding the concept being studied [58] and can help people explore and clarify their perspectives to a greater extent than is possible in individual interviews [59].

#### Study setting and sample

In Norway, mental health services are public and organized in tandem with general health services at the municipal level and specialist level. Hospitals and specialized mental health services are run by 19 health trusts owned and instructed by four regional health authorities on behalf of the state as owners [60]. The specialized mental health services system currently comprises 66 community mental health centres (CMHCs) consisting of outpatient clinics, mobile teams and inpatient wards [60].

Our study included four outpatient clinics for adults in four CMHCs. The informants were treatment personnel and leaders of teams, units or departments.

The CMHCs were invited to the study via a formal request sent to the leader. The invitations were distributed to leaders at lower levels and to treatment personnel. An overview of the participants is given in Table 1.

**Table 1:** Data collection

	Outpatient clinic no. I	Outpatient clinic no. II	Outpatient clinic no. III	Outpatient clinic no. IV
Individual interviews with treatment personnel and leaders	Psychiatrists: 1 Psychologists: 9		Leader (nurse): 1	Leader (nurse): 1
Group interviews with treatment personnel, some of whom were leaders of teams or units	Not performed	Psychiatrists: 1 Psychologists: 4 Nurses: 3 Others: 3	Psychologists: 5	Psychiatrist: 1 Psychologists: 4 Nurse: 1 Others: 3

#### Data collection

The interviews were conducted between May and November 2019 and took place face to face by one or two researchers visiting the clinic. Individual interviews lasted between 40 and 60 min, and focus group interviews lasted approximately 90 min.

A partially structured interview guide was used. The informants were asked about their attitudes towards and experiences with the pathway system and the implementation process as well as how the system influenced their everyday work.

All interviews were audio-recorded and transcribed.

#### Data analysis

The interview transcripts were first read in their entirety and were later imported into NVivo qualitative software. The data were analysed utilizing systematic text condensation [61], where codes were created based on the data and were concentrated on the main themes from the interview, namely, positive and negative expectations regarding care pathways, negative and positive experiences with the implementation process, and experiences concerning the pathways’ influence on the informant’s everyday work practices. Codes were subsequently clustered to form descriptive themes, for example, “increased time spent on coding work processes.” Furthermore, the descriptive themes that were related were clustered together to form analytical themes, for example, “care pathways lead to increased administrative work.” All included themes were grounded in the text throughout the analysis.

## Results

“There is something problematic about the fact that we are asked to do a whole lot of extra things, including more coding, more questions, more evaluation and stuff, without anything being added. We need to learn a whole new way of organizing our work, new systems, new codes, in addition to everything else we are working on. I don’t understand how we are going to make it work…” (psychologist)

The results indicated four distinct themes or reactions towards the care pathway system. These themes were lack of clarity regarding the overall goals and content of the care pathway; new codes, registration, and deadlines in the care pathway; an IT and journal system that did not correspond to the care pathway; and last, interference of the care pathway with discretion and autonomy, creating some professional dilemmas.

### Lack of clarity. “*Care pathways, what are they?”*

*“*We didn’t really know what the care pathway was, and we spent some time figuring out what it was, but when we asked our leader, we were told to await.” (psychologist)

The implementation strategy and start date were postponed and changed several times during the care pathway’s birth and infancy. This had ripple effects on the rollout within the different clinics and, as one psychologist stated,

*“*There has been so much talk about the visions and goals behind the care pathway, why it is so important. But what does it look like in the clinic? No one really knows, it seems…”

This uncertainty was handled by the leaders by telling their staff to await further action, as one clinical leader said, *“*The pathway, well, I don’t like it at all, I must say. We already have too much to do. However, I try not to show this to the staff, so when they ask me about it, I just tell them to await things.”

However, awaiting the next step created unrest within the clinic but at the same time allowed everyday work to continue.

The care pathway’s overall goal of improved quality within the mental health services e.g., increased user participation and better coordination, were relatively open for interpretation in terms of their attainment. The leaders of the outpatient clinics were responsible for implementing the care pathway. Despite the efforts made by some of the leaders to involve the staff in the overall goals, the most common strategy for the professionals was to ignore further involvement with the overall goals, because as many stated, “This is something we are already doing and have been doing for many years.”

### Codes, registration, and deadlines in the care pathway

“… None of these codes are anchored on how mental health work actually takes place. Treatment is difficult to plan, because the effect of treatment is unpredictable.” (psychologist)

Many professionals thought that the workflow presented in the pathway system and the codes involved did not correspond to a real-world timeline for mental health patients. Moreover, statistics based on the codes registered – for example, when deadlines are not met – could be traced to the therapist’s work, without attention to all kinds of reasons behind the codes, e.g., patient no-show, holidays, rotation in inpatient wards, or access to a specialist to make a clinical decision. All these elements caused stress by imposing a rigid time system without taking into consideration that breaks often occur and are more generic than the pathway system accounts for. A psychologist expressed the following opinion that was shared by many of our informants:

“The deadlines between action points are way too short. I often see that I have negative time breaks that do not count as legitimate time breaks [in the coding system], so I'm punished for that.”

The timeline and following deadlines therefore did not reflect work as it unfolds within an outpatient clinic. The consequences of this limitation were an overall feeling of frustration towards the system and the opinion that the idea behind the pathway was for the government to be more in control of work within an outpatient clinic.

*“*All this coding and administration, everything that is involved with the care pathway is just based on an idea that the government does not trust us or understand what we are doing. They want to control us.”

### An IT and journal system that does not correspond to the care pathway

*“*All these new deadlines are supposed to be coded, however through an IT system that does not correspond with the new coding. So, everything needs to be written down and remembered. I mean, what's the point?” (psychologist)

Registration of the action points, such as providing a diagnosis or evaluations within the care pathway, became a problem for several reasons. The lack of anchoring for real-time usage was vital; however, this was not the only issue. Another problem was that the different electronic journal systems used in the clinics did not correspond completely to the new codes. The practical implications were that deadlines were followed manually one way or another by the therapist, for example, by keeping an account for each patient. The frustration this caused was immense.

For practitioners with many patients, this meant a large amount of extra work, as this psychologist explains:

*“*I have 25 patients at any time, and the computer system does not tell you about the deadlines, so we need to write it down in a paper book that we are told not to use, and in addition I need to remember it, so I get quite stressed about it…”

In addition to extra work, this manual “book-keeping” – which could be done using an Excel sheet or the therapist’s Filofax – also caused stress due to privacy concerns. A more comprehensive and overarching problem that required sensemaking was that the ideal workflow for a care pathway interfered with professional values of discretion and autonomy. This will be further elaborated in-depth in the final section.

#### Care pathway interference with discretion and autonomy: when standardization creates professional dilemmas

*“The relation between patient and provider is the most important factor when it comes to healing. This means creating a space of trust where the patient decides what to share and when to share it. Some of the questions (from the care pathway could actually make patients more sick by retraumatizing them…” (psychologist)*

The pathway involves a distinction between the assessment period and the treatment period, with a deadline of six weeks to finish the assessment and give the patient a diagnosis. Many professional dilemmas related to this timeline were presented by health professionals. First and foremost, many providers had a negative reaction to the care pathway system’s emphasis on the use of formal schemes and standardized questions, for example, in the first meeting with the patient. It was a concern that this approach could negatively impact the relationship between the patient and the treatment provider. This relationship between the patient and provider is of particular importance in mental health care. Many professionals expressed a concern that the care pathway invaded this relation and had the potential to negatively influence patient treatment, as the psychologist quoted above explained.

Second, in addition to the deadline of six weeks to finish the assessment and the standardized manuals utilized, the care pathway operates with a separation between diagnostic practice and treatment that does not correspond to real-world work practice, as this psychologist explains:

“Important information for the diagnosis is sometimes not given before many months have passed, and the patient feels safe enough and trusts me with this kind of information. So, this distinction between diagnosis and treatment is not anchored in reality.”

Third, having the autonomy to organize treatment is of vital importance for mental health professionals. However, the care pathway has the potential to influence this autonomy by dictating that the first encounters are centred around assessment and diagnostic practice. This approach could influence professionals’ experience of autonomy, as a psychologist explained:

*"*If a patient has trouble with sleep, the care pathway states that I must wait at least four consultations before I can do something about it, because the assessment and diagnostic practice must happen first, even if the patient is obviously depressed and has major sleep issues."

Finally, the above shows that the care pathway influences the core values of mental health professionals, namely, autonomy when planning for treatment and discretion when providing treatment.

## Discussion

This study sought to elaborate on the sensemaking that health professionals experienced during the first ten months of care pathway implementation in four outpatient clinics in the Norwegian specialist mental health services system. In Møllering’s theory on trust, three elements were found to influence trust and distrust: calculated interest, familiarity, and compatible norms and values.

The analysis shows how sensemaking circulated around two cues within outpatient clinics. One cue was to await further action, and the other was to recognize that the health professionals were already doing the necessary work. Both cues led to actions of avoiding and reducing the importance of the implementation.

### Care pathways and trust

Sutcliffe [62] states that sensemaking occurs as follows: *when enacting order into the ongoing circumstances from which they extract cues, people act their way into knowing* [62].

Regarding the issues related to care pathways, the fundamental question is why the care pathway needs to be made sense of when the pathway is designed to improve the issues that are faced in mental health services. The analysis thus far has shown that ignoring the pathway is more important than actively engaging in it. Therefore, in regard to Sutcliffe and the act of knowing, an examination of the pathway’s relation to trust will help us understand why sensemaking occurs.

Elaborating Møllering’s [48] theory on trust shows how the issue of trust also depends on social norms and values, thus offering an explanation of how the pathway system was interpreted, understood, and made sense of in matters of trust and distrust. Furthermore, Møllering’s three elements of calculated interest, familiarity and compatible norms and values are of particular importance and will be further elaborated.

#### Trust and calculated interest

The pathway system is based on ideas of standardization [63, 64] and new public management [65], where increased control and efficiency are some of the guiding goals [1, 9, 65]. Thus, health professionals interpret care pathways as health authorities’ interest in having more control over the activities and development of these services.

This mistrust was expressed by the health professionals in our study as the need to defend their work practices and the amount of time spent on different procedures, as well as an overall idea of the need for control of mental health professionals. The interest is therefore calculated as a belief that the intent behind the care pathway system was not first and foremost to improve the services but rather to attain more control of the services.

#### Trust and familiarity

Familiarity is understood as the general premise that prior interaction creates “familiarity” and in turn enables organizations to develop confidence in each other’s trustworthiness [38]. The relationship between health authorities and mental health services is characterized by a general reciprocal scepticism towards each other’s intentions, something that makes implementing policy within this sector difficult [1, 9]. The context of increased control and management over the services within this field is based on several legislative changes during the last decades [66, 67], where the government is aiming for more transparency. The way the care pathway system evolved was characterized by mixed messages and a lack of a clear and coherent strategy, as seen by health professionals. This poor delivery of the new services further increased their aversion and led to distrust towards the care pathway and its developers. However, the issues of trust also had ripple effects on the professionals’ work practices, a phenomenon that needs to be understood more thoroughly.

#### Trust and compatible norms and values

Work in a mental health clinic is characterized by several elements, such as unpredictability, difficulties in planning treatment and a high degree of discretion and autonomy, because each patient needs individual care [68]. All these elements are based on strong, professional values on which treatment and care rest. First, the elements of autonomy and individuality collide with some of the intentions of the pathway system, such as efficiency, equality and standardization [68, 69]. This incongruity makes the implementation of these measures difficult, as Sutcliffe [62] explains, when the actors involved understand, judge and interpret the care pathways from a professional identity. Therefore, the elements of the pathway system that do not correspond to an individual’s professional identity are interpreted accordingly. In addition, as Calnan and Rowe [70] describe, new policies affect the organization as much as they influence trust when influencing the identities, skills, and prioritizations performed by professionals and managers [70]. Because the care pathway did not take their work practices into consideration and was grounded on other values, the skills and prioritizations performed by the professionals discredited the care pathway and led to further distrust.

All these issues explain how the pathway system required overall sensemaking in an attempt to disregard its importance within the outpatient clinic.

### Making sense of the care pathway

#### Making sense of the care pathway by avoiding it and reducing its importance

First and foremost, *sensemaking is an explicit response to chaos, which generates “an undifferentiated flux of fleeting sense impressions”* [71]. This chaos creates the need to make sense of something, and while doing so, restore the order that allows everyday work to continue. Professionals working within mental health care often deal with high workloads, a large amount of responsibility and work that is mentally demanding [72]. The care pathway system was developed in an attempt to be the solution to some of these issues. The important question therefore becomes why mental health professionals experience the pathway as a stressor. Their elaborations of how sense is made when they experience issues that cause frustration and stress [72] show how individuals look for cues to cope with the experience [56].

As the data clearly show, care pathway implementation caused frustration for the participating health providers. The results identified two main cues within these services. The first cue was *to await further action*. The second cue was to recognize that *we are already doing the necessary work*. Both cues led to an overall sensemaking conclusion that indicated that the professionals should ignore the content of the care pathway because “*plausible explanations shape sensible situations: they normalize the breach, restore expectations, and enable projects to continue”* [10]. In this way, health professionals could continue their everyday work.

#### Making sense of threats to professional values by fooling the system

An important concern among professionals in our study was that the care pathway generates issues that influence professional autonomy by dictating when professionals should provide assessment and diagnostic practice and when the treatment phase should start. More precisely, the distinction between assessment and treatment in the care pathway system, as well as the rigid manuals, have the potential to influence health professionals’ autonomy and discretion and potentially negatively influence treatment [73, 74]. Interruption of the subsequent relationship between a health professional and a patient is understood as something that potentially threatens professional mental health work [1, 9, 74]. In addition, as Sutcliffe [62] states, “*identity and identification provide clear frames of reference from which judgements and interpretations fan out”* [62].

Under these circumstances, health professionals make sense of the pathway system so that the threat to their professional identity is eliminated. The approach in an outpatient clinic is understood as the use of different decoupling mechanisms aiming to maintain professional autonomy in daily practice and meetings with new patients, e.g., continuing assessment when the patient is in the treatment phase or avoiding questions that could potentially negatively influence patient treatment. Therefore, the same actions that preserve discretion and autonomy discredit the system upon which the care pathway is built. Therefore, the resulting cue is to ignore parts of the care pathway, in line with the conclusion of the previous analysis.

## Conclusions

Despite the issues facing current mental health services and the attempt to solve some of them through the care pathway, the introduction of this system was met with much resistance. The issues of distrust from professionals working within mental health specialist services towards politicians and policy-makers responsible for different arrangements to be implemented in health care, such as standardization and evidence-based medicine, were further reinforced by the introduction of the care pathway system. Health professionals agreed with the overall goals of the care pathway system, such as greater user participation and better coordination. However, their emphases, worries and perspectives were first and foremost on what they perceived to be controversial and challenging about the system – the measures, coding and increased administrative work and what they perceived as a reduction in the time dedicated to patient assessment and treatment. We sought to determine how mental health professionals made sense of the pathways and how issues of trust affected their implementation. Our findings and analysis show that issues of trust or, more precisely, issues of distrust, affect how mental health professionals make sense of the care pathway by reducing its importance within the organization. These issues of trust have further implications, because it seems that the measures that affect distrust and resistance towards the pathway overshadow the care pathway’s overall goals, such as greater user participation and better coordination. This sentiment was indeed shared by mental health professionals. Changing professional practice within mental health care, where professionals are guided by strong professional values, has been shown to be complicated. Our study confirms this observation. Furthermore, trust between authorities and mental health care professionals in Norway probably needs to be restored for better success with top-down policy implementation.

## Data Availability

The datasets generated and/or analysed during the current study are not publicly available due to regulations provided by the NSD-Norwegian Centre for Research Data. Data are available only upon request to the corresponding author; however, additional written consent to share the data from each participant needs to be sampled. For more information, see the National Competence Centre for Data Protection and Data Management in Norway.

## Abbreviations

CP: Care pathway
NPM: New public management

## Footnotes

1 The Norwegian Directorate of Health, National plan for implementation of care pathways 2018–2020
